# Iron-Refractory Iron Deficiency Anemia

**DOI:** 10.4274/tjh.2014.0288

**Published:** 2015-02-15

**Authors:** Ebru Yılmaz Keskin, İdil Yenicesu

**Affiliations:** 1 Samsun Education and Research Hospital, Clinic of Pediatric Hematology and Oncology, Samsun, Turkey; 2 Gazi University Faculty of Medicine, Department of Pediatric Hematology, Ankara, Turkey

**Keywords:** Iron deficiency, TMPRSS6, Matriptase-2, Hepcidin

## Abstract

Iron is essential for life because it is indispensable for several biological reactions, such as oxygen transport, DNA synthesis, and cell proliferation. Over the past few years, our understanding of iron metabolism and its regulation has changed dramatically. New disorders of iron metabolism have emerged, and the role of iron as a cofactor in other disorders has begun to be recognized. The study of genetic conditions such as hemochromatosis and iron-refractory iron deficiency anemia (IRIDA) has provided crucial insights into the molecular mechanisms controlling iron homeostasis. In the future, these advances may be exploited to improve treatment of both genetic and acquired iron disorders. IRIDA is caused by mutations in TMPRSS6, the gene encoding matriptase-2, which downregulates hepcidin expression under conditions of iron deficiency. The typical features of this disorder are hypochromic, microcytic anemia with a very low mean corpuscular volume of erythrocytes, low transferrin saturation, no (or inadequate) response to oral iron, and only a partial response to parenteral iron. In contrast to classic iron deficiency anemia, serum ferritin levels are usually low-normal, and serum or urinary hepcidin levels are inappropriately high for the degree of anemia. Although the number of cases reported thus far in the literature does not exceed 100, this disorder is considered the most common of the “atypical” microcytic anemias. The aim of this review is to share the current knowledge on IRIDA and increase awareness in this field.

## Abstract (tr)

Demir, oksijenin taşınması, DNA sentezi ve hücre çoğalması gibi çeşitli biyolojik reaksiyonlar için vazgeçilmez olduğundan, yaşam için zorunludur. Demir metabolizması ve bu elementin düzenlenmesiyle ilgili bilgilerimiz, son yıllarda belirgin şekilde değişmiştir. Demir metabolizması ile ilgili yeni bozukluklar tanımlanmış ve demirin başka bozuklukların kofaktörü olduğu anlaşılmaya başlamıştır. Hemokromatozis ve demir tedavisine dirençli demir eksikliği anemisi (IRIDA; “iron-refractory iron deficiency anemia”) gibi genetik durumlar üzerinde yapılan çalışmalar, vücuttaki demir dengesini kontrol eden moleküler mekanizmalar ile ilgili önemli ipuçları sunmuştur. Bu ilerlemeler, gelecekte, hem genetik hem de kazanılmış demir bozukluklarının daha etkili şekilde tedavi edilmesi amacıyla kullanılabilir. IRIDA, demir eksikliği ile giden durumlarda, hepsidin üretimini baskılayan matriptaz-2’yi kodlayan TMPRSS6 genindeki mutasyonlardan kaynaklanmaktadır. Hastalığın tipik özellikleri, hipokrom, mikrositer anemi, çok düşük ortalama eritrosit hacmi, oral demir tedavisine yanıtsızlık (veya yetersiz yanıt) ve parenteral demire kısmi yanıttır. Klasik demir eksikliği anemisinin aksine, serum ferritin değeri genellikle hafif düşük ya da normal aralıkta; serum ve idrar hepsidin değerleri ise, aneminin derecesi ile orantısız şekilde yüksek bulunur. Şimdiye kadar literatürde bildirilmiş olguların sayısı 100’ü geçmediği halde, IRIDA’nın, “atipik” mikrositik anemilerin en sık nedeni olduğu düşünülmektedir. Bu derlemenin amacı, IRIDA hakkındaki güncel bilgileri araştırıcılar ile paylaşmak ve bu alandaki farkındalıklarını arttırmaktır.

## INTRODUCTION

Iron deficiency anemia (IDA) has been described for centuries. As a very frequent disorder, IDA constitutes a serious public health problem. It usually develops due to low intake of dietary iron; in the presence of hypochromic, microcytic anemia, it is the first underlying cause to be considered. However, some inherited conditions with variable clinical characteristics may also result in microcytic anemia by causing defective iron metabolism. Iron-refractory iron deficiency anemia (IRIDA; OMIM #206200), which was described only recently, is one such disorder [1].

Iron-refractory iron deficiency anemi develops due to mutations in TMPRSS6, the gene encoding matriptase-2. Its typical mode of inheritance is autosomal recessive, and IRIDA is characterized clinically by hypochromic, microcytic anemia with an inadequate response to oral iron and an only partial response to parenteral iron [1,2]. 

To date, over 50 TMPRSS6 mutations in individuals with the IRIDA phenotype have been reported [1,3,4,5,6,7,8,9,10,11,12,13,14,15,16,17,18,19,20,21]. The mostly normal growth and development of IRIDA patients and their almost normal hematologic findings in adulthood prevent the precise determination of IRIDA frequency. However, IRIDA is considered the most frequent disorder among both congenital iron metabolism disorders and atypical microcytic anemias [22]. We think that this review will increase awareness of this issue and facilitate recognition of cases of this autosomal recessive inherited disorder.

## History

Although the genetic basis of IRIDA was elucidated only recently, the disorder was first described clinically in the early 1980s [1,23]. Buchanan et al. reported IDA in 3 siblings who did not respond to oral ferrous sulfate therapy. Additionally, there was no history of poor dietary iron intake or gastrointestinal blood loss. None of the 3 children had any clinical or laboratory findings suggestive of a generalized malabsorption disorder. Notably, intramuscular iron dextran injection resulted in an only partial hematologic response; however, serum ferritin levels increased, indicating a rise in iron stores, although serum iron levels remained low. The authors suggested that the specific iron absorption disorder may partially explain the phenotype, and an additional disorder in iron utilization may result in the partial response to intramuscular iron. The occurrence of the disorder in the 3 siblings pointed toward the hereditary nature of the disease. Cases reported subsequently supported this observation [24,25,26,27,28]. However, 27 years passed before the genetic basis of the disease was finally elucidated.

## Pathophysiology

**Figure 1:**
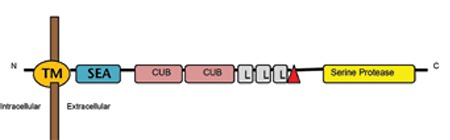
Schematic representation of matriptase-2, encoded by the TMPRSS6 gene. N: amino-terminus, C: carboxy-terminus, TM: transmembrane domain; SEA: sea urchin sperm protein, enteropeptidase agrin, CUB: complement protein subcomponents C1r/C1s, urchin embryonic growth factor and bone morphogenic protein 1 domain, L: low density lipoprotein receptor class A domain (LDLR), Serine Protease: serine protease domain, red triangle: cleavage activation site.

Iron-refractory iron deficiency anemia develops due to mutations in TMPRSS6, which is the gene encoding matriptase-2. Matriptase-2 is a type II transmembrane serine protease expressed mainly in hepatocytes (Figure 1) [1,3,29]. It plays a role in downregulating hepcidin expression in liver cells. TMPRSS6-mutant mice were found to have microcytic anemia and elevated hepcidin levels disproportionate to the degree of anemia [30].

Similar to the findings in TMPRSS6-mutant mice, the hepcidin levels in serum, plasma, and urine samples of IRIDA patients were found to be either within or above the normal ranges of those in healthy adults [1,3,4]. In classic IDA, hepcidin levels decrease markedly to promote intestinal iron absorption [31,32]. Thus, normal-to-elevated hepcidin levels observed in IRIDA patients reflect the inability to appropriately regulate this protein.

Hepcidin, the master regulator of iron metabolism, is a small peptide synthesized in the liver. In cases of iron loading, hepcidin expression is induced to inhibit intestinal iron uptake, whereas iron deficiency results in reduced hepcidin expression to increase the availability of iron for erythropoiesis [33,34]. Hepcidin exerts its iron regulatory effects by binding to ferroportin (a cellular iron exporter expressed on the basolateral membrane of enterocytes) on the plasma membrane of macrophages and in hepatocytes. Ferroportin is the only known cellular iron exporter. Because hepcidin binding leads to the internalization and degradation of ferroportin in lysosomes, dietary iron absorption and mobilization of iron from macrophage stores are decreased if hepcidin levels are elevated [35,36,37,38]. Therefore, the decrease in ferroportin expression through hepcidin can explain the development of iron deficiency and unresponsiveness to oral iron in IRIDA cases.

Before it can be utilized in erythropoiesis, parenteral iron administered in the form of iron-carbohydrate complexes must be processed and exported by macrophages. This export step is also ferroportin-dependent. Therefore, inappropriately high hepcidin levels can additionally explain the sluggish and incomplete response to parenteral iron observed in IRIDA cases.

**Figure 2:**
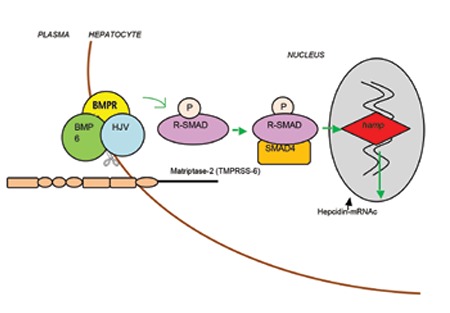
Schematic model of hepcidin regulation by matriptase-2. BMP: bone morphogenetic protein, BMPR: BMP receptor, HJV: hemojuvelin, R-SMAD: receptor-associated Son of Mothers against Decapentaplegic proteins. In conditions of iron deficiency, matriptase-2 modulates hepcidin signaling by cleaving HJV from the hepatocyte plasma membrane, resulting in decreased hepcidin production.

In a cell-model study, matriptase-2 was found to regulate hepcidin expression by cleaving hemojuvelin (HJV), a protein found on the plasma membrane of hepatocytes, to promote the expression of hamp, the gene encoding hepcidin. Therefore, the final effect of matriptase-2 is attenuation of hepcidin activation (Figure 2) [39]. HJV promotes hepcidin expression through a pathway involving bone morphogenetivc proteins (BMPs). Following the interaction of BMPs with their specific receptors on the cell surface, sons of mothers against decapentaplegic (SMAD) proteins become phosphorylated owing to the serine/threonine activity of BMP receptors. SMAD proteins are a class of intracellular signaling molecules. Following phosphorylation, SMAD proteins bind to SMAD4, a common mediator. The heteromeric complexes formed translocate to the nucleus and regulate the transcription of target genes, among which is hamp [40,41]. Therefore, loss of matriptase-2 activity caused by TMPRSS6 mutations results in hepcidin overexpression.

In healthy fetuses and neonates, because of the rapid growth and expansion of the red cell compartment, hepcidin gene expression is drastically repressed [42,43]. However, the role of matriptase-2 in this repression was not elucidated until recently. In their very recent study, Willemetz et al. found that in TMPRSS6-/- fetuses, liver Hamp1 mRNA expression was up to 60 times higher than that in control mice, in which hepcidin expression was only barely detectable [44]. It was noteworthy that TMPRSS6-/- fetuses and newborns had lower iron content, mean corpuscular erythrocyte volume (MCV), and hemoglobin (Hb), indicating microcytic anemia secondary to iron deficiency in mutant mice. However, the red blood cell (RBC) levels were unaffected. These observations suggest that the requirement of matriptase-2 for hepcidin suppression begins in utero, and its deficiency leads to microcytic anemia.

Despite the laboratory findings of iron deficiency in individuals with TMPRSS6 polymorphisms or mutations suggested to be mediated by the inability to suppress hepcidin expression appropriately, 2 population-based studies have found that the affected iron and erythrocyte parameters are at least partially independent of hepcidin levels [45,46]. Similarly, Lehmberg et al. reported that the urinary hepcidin levels of patients with the IRIDA phenotype and TMPRSS6 mutations were either below the normal range or undetectable [19]. These observations suggest that, in the presence of TMPRSS6 mutations, underlying mechanisms other than the inability to downregulate hepcidin expression may be responsible for the laboratory findings of iron deficiency. Studies to elucidate those mechanisms are underway.

## Clinical Presentation

Iron-refractory iron deficiency anemia can present with various clinical and laboratory characteristics. Similarly, there is a discrepancy in the response to treatment among patients. However, individuals with IRIDA are usually diagnosed during childhood. Patients most commonly present with mild to moderate anemia, and their growth and development are normal. The key features of the disease are: 1) congenital hypochromic, microcytic anemia; 2) very low MCV (patients present with marked microcytosis and hypochromia that are disproportionate to the degree of anemia); 3) low transferrin saturation; 4) abnormal iron absorption; 5) defective iron utilization (as evidenced by sluggish and incomplete response to parenteral iron); and 6) an inheritance mode compatible with autosomal recessive transmission [1]. When laboratory findings of the IRIDA cases reported to date are reviewed, it can be noted that anemia was detected in the first blood count testing performed in most of the individuals (children); this testing usually occurred before the age of 2 years. Because the subjects are usually healthy children with normal growth and development, blood count testing was typically not ordered due to a specific indication but was rather performed as a part of routine screening. The recent findings of Willemetz et al. in TMPRSS6 knockout mice may shed light on the beginning of laboratory findings associated with IRIDA. They suggest that (mild) microcytic anemia may actually be present in utero and at birth in IRIDA cases [44].

Recently, de Falco et al. reported the findings of a Turkish female infant who had a molecular diagnosis of IRIDA (testing was ordered because of a family history of IRIDA) at the age of 3 months before she developed an overt IRIDA phenotype [47]. The physical examination findings of the infant were normal at birth, with the birth weight being appropriate for the gestational age. The follow-up data of the same infant were later reported in another study, which stated that findings of a typical IRIDA phenotype were obvious when she was 4 months old [21].

In the evaluation of the cases regarding treatment, a temporary rise in serum ferritin was observed after (parenteral) iron therapy [3,4,8,21,48]. Once administered, intravenous (i.v.) iron in a colloidal form (iron gluconate) enters reticuloendothelial cells [49,50]. Following sequestration of iron in these cells (as evidenced by an increase in the serum ferritin level), a portion of the iron probably reaches plasma transferrin despite high hepcidin levels and can be utilized in erythropoiesis; this results in partial correction of anemia and a slight increase in MCV. Indeed, the uptake of colloidal iron by reticuloendothelial cells results in an increase in the serum ferritin level; however, upon binding of iron to transferrin in the plasma, both the iron load in macrophages and the serum ferritin concentration gradually decrease again.

In their study including 11 children with IRIDA, Akin et al. reported their findings following i.v. iron administration [51]. The Hb and serum ferritin levels of the patients increased to 9.5 g/dL and 24 ng/mL, respectively, at 6 weeks after the first therapy. Although the level of Hb was steady, ferritin levels continued to increase up to 30 ng/mL and 47 ng/mL at 6 months after the first week and 6 weeks after the second therapy, respectively. Thus, the authors suggested that i.v. iron should be administered only once in IRIDA cases because its continued administration would be of no benefit in terms of increasing Hb levels. Additionally, in their study, Khuong-Quang et al. reported 2 siblings who presented with very high ferritin levels at admission in the absence of iron treatment [20]. These observations point to the variability of the genotype-phenotype correlations in IRIDA cases [52].

Unresponsiveness to oral iron therapy is considered one of the hallmarks of IRIDA; however, some recent studies have reported the correction (at least partially) of hematological parameters after prolonged and/or high-dose oral iron therapy [12,16,20,21,53]. With high-dose oral iron (6-10 mg/kg/day elemental iron) for up to 17 months, acceptable Hb levels were achieved in some of these cases, although microcytosis, hypoferremia, and low transferrin saturation persisted in most of the cases. Recently, a 5-month-old Sardinian female infant with a homozygous TMPRSS6 mutation who was unresponsive to oral iron and partially responsive to i.v. iron displayed a marked increase in the Hb level (up to 121 g/L) following the use of the combination of oral iron and ascorbic acid for 3 months [54]. If confirmed in more patients, this combination treatment may offer an alternative in the treatment of patients with IRIDA and simplify their management.

Because of the paucity of IRIDA cases reported to date, data concerning the clinical course and long-term follow-up of these individuals are limited. Nevertheless, it has been observed that the low Hb levels in early childhood increased to acceptable values in adulthood in those few cases that could be followed [3]. However, some of the laboratory findings indicating iron deficiency (low MCV, mean corpuscular Hb [MCH], serum iron, and transferrin saturation) had persisted. Notably, the ferritin levels of these patients tended to increase with age. Because iron is needed during childhood for body growth, particularly for red cell mass expansion, the less severe anemia phenotype in these individuals in adulthood was explained by the consequence of the greater availability of the limited amount of dietary iron for erythropoiesis.

## Genetics

IRIDA was first associated with a genetic locus on the long arm of chromosome 22 (22q12.3-13.2) in the Sardinian family members reported by Melis et al. [3]. In the affected individuals, the disorder could be attributed to a mutation in the homozygous state arising in a common ancestor. Subsequently, other families with the IRIDA phenotype were evaluated and a recessive mode of inheritance arising from mutations in the same genetic locus was confirmed [1]. Included within the critical chromosome 22 region was the gene TMPRSS6, which encodes matriptase-2, a protein belonging to the type II transmembrane serine protease family. This group contains a short cytoplasmic amino terminal tail, a transmembrane region, a stem region with several structural domains, and a carboxy-terminus serine protease domain (Figure 1) [29,55].

IRIDA can be found in individuals from a range of ethnic backgrounds; to date, there is no evidence for a significant founder effect. All of the reported mutations are predicted to cause functional loss in the encoded protein. Most of them are missense mutations; however, frameshift, intronic, and nonsense mutations, as well as one large in-frame deletion, have also been reported (Tables 1 and 2) [1,3,4,5,6,7,8,9,10,11,12,13,14,15,16,17,18,19,20,21]. The mutations are spread throughout the gene sequence, and they disrupt not only the serine protease catalytic activity but also other domains participating in protein-protein interactions [1,3,4,5,6,10,11,16]. In vitro studies have demonstrated that causative TMPRSS6 mutations are associated with reduced inhibitory activity on the hepcidin promoter compared with the wild-type proteins [5,6,10].

In the parents of IRIDA patients with TMPRSS6 mutations, as expected, normal erythrocyte and serum iron parameters were reported because the typical transmission mode of the disorder is autosomal recessive. However, in some family members of individuals with IRIDA, iron deficiency was reported under certain clinical conditions, an observation inconsistent with recessive transmission. As an example, Hartman et al. reported intramuscular iron use in the maternal aunt of 2 affected siblings during pregnancy and the persistence of iron deficiency even after hysterectomy. Additionally, she responded poorly to an oral iron absorption test [26]. We were also informed of anemia worsening in the mothers of our patients with IRIDA (unpublished data). These women were all heterozygous carriers of a pathogenic TMPRSS6 mutation, and at times other than during pregnancy, they had acceptable complete blood count results. Similarly, the maternal grandmother of 2 children with IRIDA was reported to have required regular intramuscular iron therapy throughout adulthood [24]. Supporting these observations, TMPRSS6-haploinsufficient mice were found to be more susceptible to iron deficiency under conditions of iron restriction or an increased iron requirement, such as pregnancy [56,57]. However, it remains unclear whether certain environmental factors, a low-expressing allele, a combination of modulating polymorphisms, or defects in other genes can explain this observation.

Common genetic variants such as single-nucleotide polymorphisms (SNPs) in the TMPRSS6 gene in several populations have been associated with changes in erythrocyte and iron parameters, such as the Hb level, MCV, MCH, serum iron level, and transferrin saturation [56,57,58,59,60]. TMPRSS6 SNP rs855791, which shows the strongest association with these parameters, is characterized by a missense change in the serine protease domain. This variant was found to be less effective in suppressing hepcidin levels in vitro and was shown to influence serum iron parameters in healthy individuals [56]. Another recent study evaluated the predisposition to IDA in the Chinese population and found 2 TMPRSS6 polymorphisms (rs855791 and rs4820268) to be genetic risk factors for iron deficiency and IDA [61].

Interestingly, Nie et al. recently reported a 10-year-old Chinese female with a triallelic polymorphism of TMPRSS6 (homozygous for c.757 A>G and heterozygous for c.2207 T>C [rs855791]), who presented with severe hypochromic, microcytic anemia (Hb 58 g/L) at the age of 15 months [62]. The patient had laboratory findings consistent with IRIDA, including inappropriately high hepcidin levels, and was unresponsive to both oral and i.v. iron. The c.757 A>G polymorphism in the homozygous state was previously reported in a 27-year-old Japanese female with mild anemia (Hb: 108 g/L) [63]. The identification of a triallelic polymorphism resulting in an IRIDA phenotype is unique to the case reported by Nie et al.

Despite these observations, current guidelines recommend IRIDA due to TMPRSS6 defect to be diagnosed with certainty only when the subject is homozygous or compound heterozygous for pathogenic TMPRSS6 mutations [59].

## Differential Diagnosis

In an individual with hypochromic, microcytic anemia, acquired causes of iron deficiency or congenital causes, mainly thalassemia syndromes, are the most common underlying pathology. Upon confirmation of iron deficiency, the next step is evaluation of acquired causes of iron deficiency, such as poor dietary iron intake, on-going blood loss, and chronic inflammatory conditions. In an untreated patient with iron deficiency, 2 initial laboratory patterns may suggest an IRIDA diagnosis: 1) very low MCV (range: 45-65 fL) relative to the degree of anemia (Hb range: 60-80 g/L); and 2) marked hypoferremia and low transferrin saturation (usually <5%) in the presence of a slightly low or even normal serum ferritin level.

Subjects with iron deficiency will usually be treated with therapeutic doses of oral iron. A poor or absent response to therapy is most commonly associated with poor compliance to treatment, inadequate dosing, or duration of therapy. If these conditions are avoided and adequate oral iron is taken by the patient, the absence of a hematologic response should indicate first a possible defect in iron absorption. Among the causes of poor iron absorption are achlorhydria and duodenal damage, such as celiac sprue. In addition, because hepcidin production is not solely dependent on iron stores in the body but also on inflammatory stimulants [34,64], both anemia of chronic inflammation (ACI) and IRIDA are associated with hepcidin elevation and result in impaired export of iron from duodenal enterocytes into the plasma. In ACI, inflammatory cytokines stimulate hepcidin expression, resulting in a decline of iron mobilization from its stores to be used in erythropoiesis [65]. Therefore, serum iron levels are found to be low in ACI, a condition that also occurs in IRIDA. However, because ACI is an acquired disorder of iron utilization, iron stores are typically normal or elevated [66], while in IRIDA, a true systemic iron deficiency state is present due to the life-long defect in intestinal iron absorption.

If oral iron challenge results indicate poor iron absorption and the onset of iron deficiency is in infancy or childhood, IRIDA is the likely diagnosis. Today, the only diagnostic test for IRIDA is genetic analysis of the TMPRSS6 gene for causative mutations. In fact, measuring serum or urinary hepcidin levels assists in distinguishing IRIDA from classic IDA. However, no approved hepcidin assay is available yet for routine clinical use. Once a hepcidin assay becomes available, the diagnosis of IRIDA may be simpler.

Hereditary iron metabolism disorders other than IRIDA may also cause hypochromic, microcytic anemia. Among these are divalent metal transporter 1 (DMT1) deficiency, congenital hypotransferrinemia, some hereditary forms of sideroblastic anemia, and aceruloplasminemia. Certain clinical and laboratory characteristics of these disorders allow their differentiation from IRIDA (Table 3) [2,59].

## Conclusion

The prevalence of IRIDA seems to be more frequent than predicted and more heterogeneous than previously thought, indicating that the “classical” severe homozygous form may in fact be just one of several forms of the disorder, with some cases of mild microcytic anemia actually also belonging to this disorder. A few points may help in a “classical” IRIDA diagnosis in the clinical practice of hematologists and pediatricians: if present, the familial nature of the disease; the presence of atypical iron parameters not in accordance with classical IDA (such as low-normal or normal serum ferritin levels accompanying very low transferrin saturation); and the absence of the expected hematologic response after the use of oral iron. If there is a high suspicion of IRIDA, the diagnosis can be confirmed with demonstration of the mutations in the TMPRSS6 gene, testing that is currently available only at some specialized laboratory centers.

The acceptable Hb levels of IRIDA patients in adulthood and normal growth and development during their childhood make the recognition of these individuals difficult. The long time interval that passed before the establishment of an IRIDA diagnosis in the cases followed supports this situation. These patients are mostly diagnosed after very detailed, and sometimes invasive, examinations. This condition can generally be proven easily considering the knowledge in the literature. In spite of the paucity of IRIDA cases reported so far, this disorder may in fact be more common in countries with frequent consanguineous marriages like Turkey, in which the rate of consanguineous marriages was reported as 22% [67]. Therefore, an increased awareness of the clinical and laboratory characteristics of IRIDA is important in such populations, particularly in terms of reducing the number of unnecessary (and possibly invasive) examinations.

## Figures and Tables

**Table 1:**
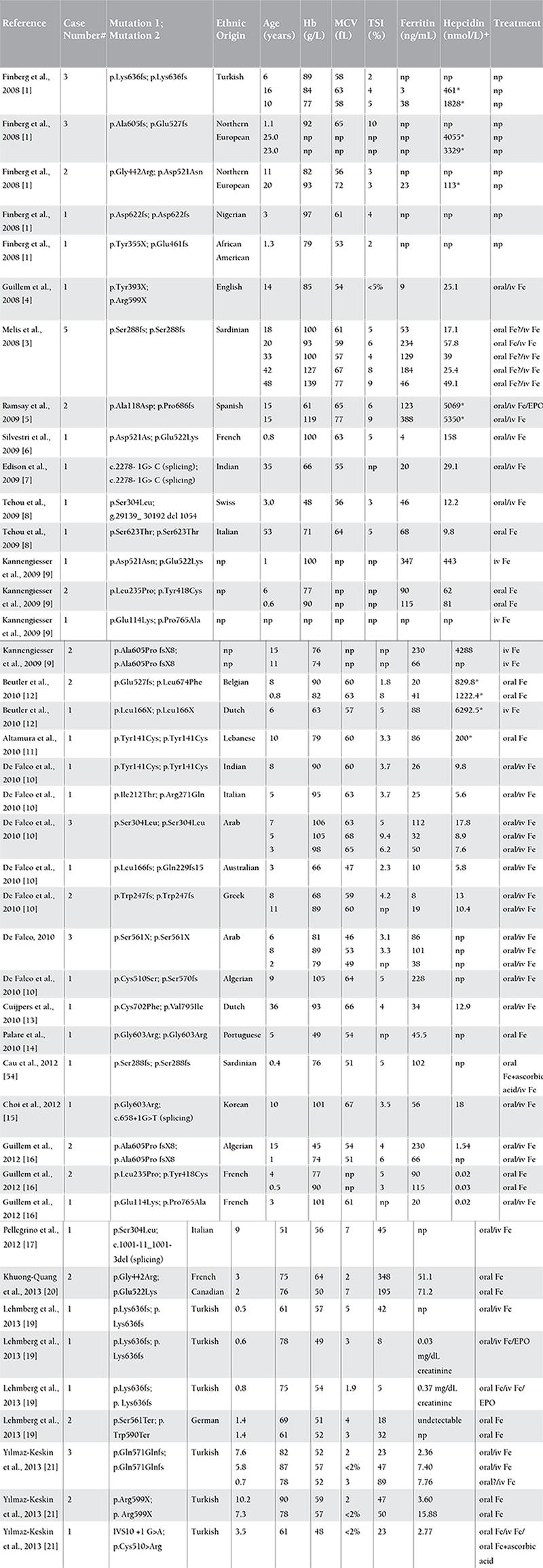
Literature review of iron-refractory iron deficiency anemia cases with homozygous or compound heterozygous TMPRSS6 mutations.

**Table 2:**
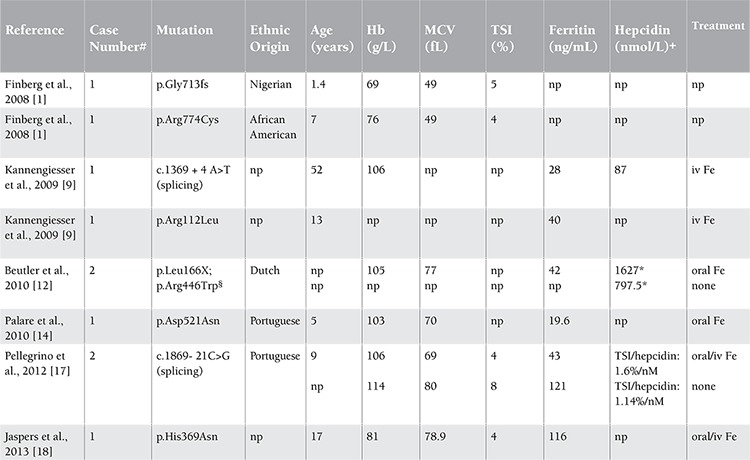
Literature review of iron-refractory iron deficiency anemia cases with a heterozygous TMPRSS6 mutation identified.

**Table 3:**
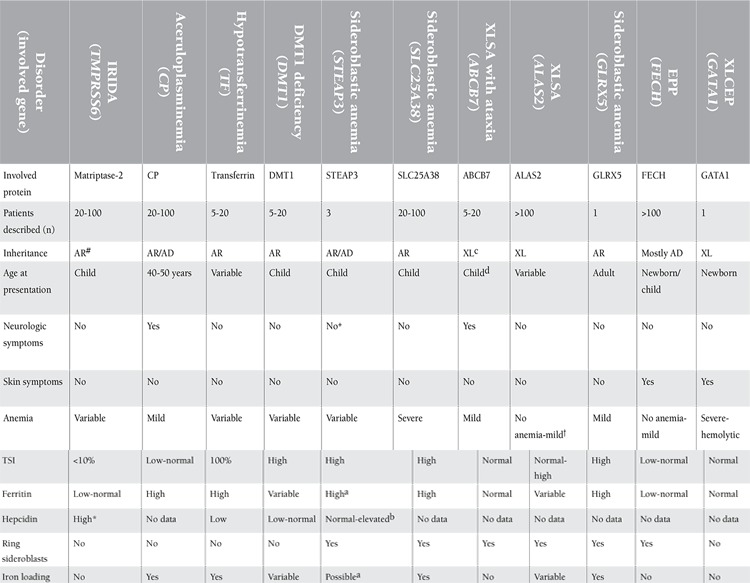
Main characteristics of rare microcytic anemias secondary to inherited disorders of iron metabolism or heme synthesis.
